# High Efficiency and Problems of Chemiluminescence Assay-Detected Aldosterone-To-Renin Ratio in Practical Primary Aldosteronism Screening

**DOI:** 10.1155/2020/3934212

**Published:** 2020-08-27

**Authors:** Wenbin Lin, Yuzhe Li, Dubo Chen, Zhenrong Yao, Hongxu Xu, Yonghong Chen, Jiahao Xiao, Pinning Feng, Wenjia Gan

**Affiliations:** ^1^Department of Clinical Laboratory, The First Affiliated Hospital of Sun Yat-Sen University, Guangzhou, China; ^2^Department of Clinical Laboratory, The Third Affiliated Hospital of Sun Yat-Sen University, Guangzhou, China; ^3^Department of Clinical Laboratory, The Sixth People's Hospital of Longgang District, Shenzhen, China; ^4^Reproductive Medicine Center, The Third Affiliated Hospital of Sun Yat-Sen University, Guangzhou, China

## Abstract

Primary aldosteronism is a main cause of secondary hypertension which can be effectively treated. The screening test for primary aldosteronism is benefit for minimizing damage to the patient. In the previous retrospective study, we obtained the optimal cutoff value of aldosterone-to-renin ratio detected by chemiluminescence assay, a newly developing method, and prompted its high efficiency in primary aldosteronism screening in upright position. In this study, we want to evaluate its efficiency in practical work. We used this ratio to continuously screen 238 patients, and 58 patients were finally diagnosed with primary aldosteronism. We found it had 86.13% accuracy rate in the upright position compared with the final clinical diagnosis. False negative and positive rates were 13.79% and 13.89%. Diagnostic sensitivity and specificity were 86.21% and 86.11%, which are slightly different from results in our previous study. False negative rate can be improved by combining the aldosterone-to-renin ratio with aldosterone concentration. We also found impaired glucose tolerance may be a reason for high false positive rate. Besides, chemiluminescence assay may be interfered in aldosterone detection. Although it has some shortcomings, chemiluminescence assay-detected aldosterone-to-renin ratio is a highly effective index for screening primary aldosteronism in practice.

## 1. Introduction

Primary aldosteronism (PA), which accounts for 5% to 20% morbidity in resistant hypertension in different reports [1–3], is a main cause of secondary hypertension [4]. It is caused by excess aldosterone secretion from one or both of adrenal glands. Excess aldosterone not only causes hypertension through sodium and water retention but also directly induces damage to vital organs, like the heart, kidney, and vasculature through inflammation, fibrosis, and tissue remodeling. Thus, PA patients are more likely to have arrhythmia, ventricular hypertrophy, cerebral infarction, renal insufficiency, etc. PA can be effectively treated by blocking aldosterone or hyperplastic adrenal gland resection [5]. So, effectively discovering disease has great significance for PA patients.

The aldosterone-to-renin ratio (ARR) is widely used to screen out PA from secondary hypertension. Results from a multicenter study show that ARR application raises 10–15 times PA diagnosis rate [2] and has been encouraged by the Endocrine Society Guideline [6]. In recent decades, chemiluminescence assay (CLIA) is applied for aldosterone and renin detection, which is a higher safety profile than the previously used radioimmunoassay (RIA) [7, 8]. Although it is recommended in aldosterone and renin detection, CLIA is not extensively used in PA diagnosis, because of the controversies in the cutoff value and diagnostic efficiency of CLIA-detected ARR [7, 9–11]. Therefore, more evidence on these two areas should be collected.

In our previous study, we determined the optimal cutoff value and diagnostic efficiency of CLIA-detected ADRR in PA screening by retrospective analysis [12]. We found that CLIA-detected ADRR has the highest diagnostic efficiency, especially specificity and positive-predictive value, when the cutoff value is 28 in the upright position. But, the ARR cutoff value for PA screening is recommended as 30 or more in other studies [13–15], which is larger than our cutoff value. We wonder whether our cutoff value is suitable for the practical PA screening. In this study, we evaluate the efficiency of our ADRR cutoff value in PA screening through a high-quality perspective study. Our results confirm the conclusion in the previous study. Meanwhile, we discover some interfering factors in practical use. Our study may attribute to the widespread application of CLIA-detected ADRR in PA screening.

## 2. Materials and Methods

### 2.1. Patients

We sequentially collected data from patients with refractory hypertension to the First Affiliated Hospital of Sun Yat-sen University (Guangzhou, China) between October 2018 and March 2019. Refractory hypertension was regarded as hypertension without remission after enough treatment according to the current guidelines. ADRRs of eligible patients were detected immediately after admission. Final diagnosis was recorded after the patient leaving the hospital. PA diagnosis was according to the guideline about PA diagnosis and treatment, which was published by the American Association of Clinical Endocrinologists in 2016 [6] and PA diagnosis standards in China. Briefly, PA diagnosis was required to satisfy following conditions: (1) typical clinical features, like hypertension and hypokalemia; (2) adrenal hyperplasia or adenoma in imaging; (3) histopathology; and (4) approval test confirmation (aldosterone concentration over 100 pg/ml in the saline infusion test or renin concentration decreased less than 30% in the captopril test). The case included in the following study should at least have a complete ADRR in the upright position and a clear PA or non-PA diagnosis.

### 2.2. Sample Collection and Detection

This study was approved by the Medical Ethics Committee of the hospital, and the requirement of written informed consent was remitted by the Medical Ethics Committee. According to guidelines, patients were asked to withdraw all antihypertensive drugs at least 2 weeks before blood collection. During drug withdrawal, the patients were carefully looked after. Drugs without influence on aldosterone and renin concentrations, like diltiazem, doxasozine, and verapamil, were used as escape medication in case of severe discomfort and/or extreme blood pressure (≥180/110 mmHg). Blood sample was collected after a full night's sleep (>8 h). After that, patients were asked to stand or walk for 2 h and seat for 15 min. Then, blood sample in the upright position was collected before 9 : 00 AM on the same day. The specimen was collected in EDTA-K_2_ anticoagulant tube. Plasma was separated by centrifugation at room temperature, 3,000 g for 5 min. CLIA kits and detecting instrument (Antu Biotech Co., LTD, Zhengzhou, China) were used to detected aldosterone and renin concentrations in plasma, which followed the manufacturer's instruction. The analytical imprecision of CLIA-detected aldosterone and renin both was less than 5%. The measuring ranges of CLIA-detected aldosterone and renin were 10–1000 pg/ml and 4–500 pg/ml. The reference intervals of aldosterone and renin in our lab were 40–310 pg/ml and 4–38 pg/ml, respectively. Corresponding aldosterone and renin concentrations were used to calculate ADRR. ADRR >28 was considered as positive for PA screening [12].

### 2.3. Statistical Analysis

SPSS v22.0 (IBM) was used in the whole statistical analysis. Quantitative data were expressed as mean value ± standard deviation (SD) or indicated. Final clinical diagnosis was considered as the gold standard, and the accuracy rate, false positive and false negative rates, sensitivity, and specificity of PA screening were calculated and evaluated. Quantitative data were tested two-sided by Student's *t* test. If normal distribution was not met, the Mann–Whitney *U* test was used. Qualitative data were tested by the nonparametric test. *p* < 0.05 was defined as statistically significant difference. The area under receiver operating characteristic curve (AUC) and Youden index were used to assess the diagnostic efficiency. The Youden index was defined as sensitivity plus specificity minus 1.

## 3. Results

### 3.1. Baseline Characteristics

Between October 2018 and March 2019, a total of 250 patients visited the hospital due to refractory hypertension. Among them, 5 patients failed to obtain complete ADRR in upright position, and 7 patients left hospital without definite diagnosis. 238 cases met our requirements at the end. Baseline characteristics of the rest 238 patients are shown in Table 1. Age and gender compositions between PA and non-PA groups had no statistical difference. 24.37% cases were clinically diagnosed as PA. Aldosterone and renin concentrations and ADRR were statistically different between two groups, which meant aldosterone concentration and ADRR in the PA group were higher than those in the non-PA group, and the renin concentration was converse. Among 58 PA patients, 24 cases had aldosteronoma only, 11 cases had idiopathic hyperaldosteronism, 9 cases had adrenal cortical hyperplasia only, 2 cases had aldosteronoma and hyperplasia both, and the rest 12 cases were unidentified.

### 3.2. Screening Efficiency Evaluation

**Figure 1:**
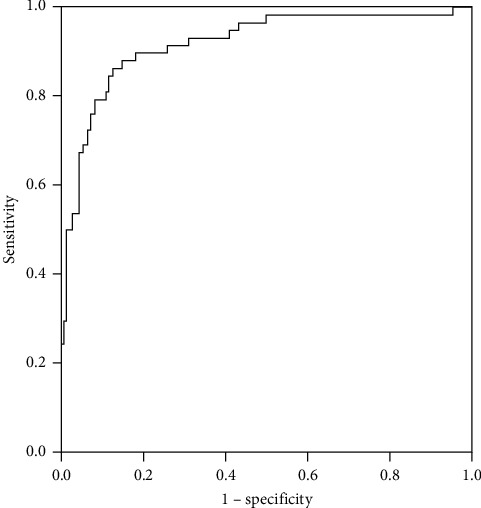
Receiver operating characteristic curve of ADRR for PA screening.

In the 238 cases, 75 cases with ADRR >28 were considered as positive in the screening test, and 50 cases were finally diagnosed as PA in the ADRR positive group, while 163 cases were negative in ADRR screening and 8 cases were finally diagnosed as PA in the group, which meant the false positive and negative rates were 13.89% (25/180) and 13.79% (8/58), respectively. The descriptive representation of 8 false negative cases is shown in Table 2. ADRR correctly screened 155 negative and 50 positive cases in this study with an accuracy rate of 86.13%. Diagnostic sensitivity and specificity were 86.21% (50/58) and 86.11% (155/180). AUC of ADRR screening in the upright position was 0.918 (0.874–0.963, 95% confidence interval) (Figure 1). We also calculated the diagnostic values of different ADRR decision thresholds for PA screening, which are shown in Table 3.

## 4. Discussion

In our previous retrospective study, we found the most effective cutoff value of CLIA-detected ADRR in the upright position was 28 for PA screening, but we did not know its efficacy in actual work. So, we evaluated its efficacy in this prospective study. The number of patients in case and control groups were estimated from the sensitivity and specificity of ADRR screening test calculated in our previous study. But, we found the number of control group could not be calculated, because the specificity was 100%. So, we took those cases with a final non-PA diagnosis as the control group which were collected at the same time as the case group. Finally, we totally had 238 cases in this study. The ratio of PA patient was 24.37%, which is close to the same ratio, 21.9% (*p*=0.434), in our previous study [12] and other studies [14, 16]. Then, we compared baseline characteristics between these two studies. There was no statistical significance in age and gender compositions, which suggests there is no significant change in age and gender compositions of refractory hypertension patients to the hospital in recent years (*p*=0.065 and 0.109). But, there was statistical significance in ADRR, aldosterone, and renin concentrations, and the main difference originated from the non-PA group, which meant aldosterone concentration and ADRR were higher in this study, but renin concentration was just the opposite. Raised ADRR in the non-PA group may account for high false positive rate and low specificity, which will be discussed subsequently.

Then, we calculated the accuracy rate. It confirms the high efficiency of ADRR for PA screening in practical work. In this study, we also calculated the sensitivity and specificity rates of ADRR for PA screening. Compared with previous retrospective study, these two rates decreased, especially specificity (100% vs. 86.11%). The lower sensitivity is due to 8 false negative cases. They can be distinguished by high aldosterone concentration (>310 pg/ml in upright position), combined ADRR in the upright and supine positions, images, etc. For example, although it increases 35 false positive cases, high aldosterone concentration recognizes 4 false negative cases in the ADRR negative group. However, the false positive case could be eliminated by many approval tests which are simple, effective. and inexpensive. So, combining ADRR with previous items is a good choice for decreasing the false negative cases. As previously described, high false positive and low specificity partially result from increased ADRR in the non-PA group. However, we do not suggest raising the cutoff value of ADRR for PA screening, because it not only decreases the false positive rate but also obviously increases the false negative rate (Table 3). It is not a good choice for PA screening, because PA can be cured. The raised false negative rate which means missing true PA patient will cause serious consequence to PA patient. Besides, the Youden index also reaches maximum when the cutoff value of ADRR is 28 in this study.

However, finding reasons for high false positive rate may help improving the screening efficiency of ADRR. To characterize false positive cases, we compared age and gender distributions between false and true positive cases, but no statistical difference is found (*p*=0.552 and 0.295), which indicates the reason for false positive may be the age and gender of patient. Then, we compared aldosterone and renin concentrations between these two kinds of cases. Although the renin concentration has no difference, the aldosterone concentration is lower in the false positive group, which suggests the false positive rate may be decrease after ADRR screening followed by selecting cases with aldosterone concentration over the reference range. But, unfortunately, it did not work well, because combination not only markedly decreased false positive cases (25 vs. 8) but also obviously reduced true positive cases (50 vs. 28). Combining ADRR screening with other items may decrease false positive rate, which needs further study.

A special false positive case also gives us some clues. Aldosterone and renin concentrations of this patient in the upright position were detected twice on different days. The first ADRR was over 28, but the second ADRR was less than 28, actually only 15.3, without any treatment. The aldosterone concentration was halved at the second test. It suggests that aldosterone may be stimulated or interfered by unidentified reason at the first test. A comparative study may verify our hypothesis. In that study, we compared the aldosterone concentration detected by CLIA and ultraperformance liquid chromatography tandem mass spectrometry. The former is almost all higher than that of the latter, and the correlation coefficient is not very high between them, which suggest CLIA is disturbed by uncertain factors. We need more accurate method to identify the interference and help us modify CLIA detection.

Diabetes is reported to raise the cutoff value of radioimmunoassay detected ARR for PA screening [17]. Then, we assessed the influence of diabetes on screening efficiency in our study. Our results suggest ADRR is influenced by not only diabetes but also impaired glucose tolerance. We found 37 and 13 cases with impaired glucose tolerance in non-PA and PA groups. Although it had no effect on ADRR in PA patients, impaired glucose tolerance elevated ADRR in non-PA patients (median, 17.99 vs. 12.32). If cases with impaired glucose tolerance were excluded, specificity and false positive rate will be improved slightly (Supplementary Table 1). It suggests a special ADRR cutoff value may be needed to screen PA from patient with impaired glucose tolerance. The incidence of impaired glucose tolerance increased in the recent years in China [18, 19], which may partially explain the declining efficiency of ADRR for PA screening in this study. Besides, metabolic syndrome is supposed to be related to primary aldosteronism [20]. Effects of other metabolic abnormalities, like hyperuricemia and hyperlipidemia, on ADRR for PA screening may also need assessment.

In this study, we assessed the efficiency of CLIA-detected ADRR for PA screening in newly enrolled patients with refractory hypertension. Overall, it is competent for making accurate primary aldosteronism screening in practical work [21]. But, it still has obvious defects, relatively high false positive and negative rates, which cause a less satisfactory accuracy rate. Further studies are needed to identify reasons and modify the performance of ADRR in PA screening. Finally, our results support the clinical application of CLIA-detected ADRR in PA screening.

## Figures and Tables

**Table 1:** Baseline characteristics.

Variable	Cohort (*n* = 238)	*p* value
Sample (cases)		
PA	58	
Non-PA	180	
Gender (cases)		0.556
Male	127	
PA	29	
Non-PA	98	
Female	111	
PA	29	
Non-PA	82	
Age (years)^a^		0.148
PA	48.59 ± 11.25 (28–72)	
Non-PA	45.83 ± 15.96 (12–86)	
Total	46.50 ± 14.97 (12–86)	
Aldosterone concentration (pg/ml)^b^		<0.001
PA	337.35 (61.52–2080.67)	
Non-PA	219.02 (55.78–990.20)	
Total	235.66 (55.78–2080.67)	
Renin concentration (pg/ml)^b^		<0.001
PA	5.55 (0.10–353.70)	
Non-PA	18.30 (2.10–146.40)	
Total	14.20 (0.10–353.70)	
ADRR^b^		<0.001
PA	70.12 (2.83–615.20)	
Non-PA	13.36 (0.97–149.72)	
Total	16.62 (0.97–615.20)	
Creatinine concentration (*μ*mol/L)^b^		0.415
PA	73.5 (42–741)	
Non-PA	71.0 (34–1677)	
Total	71.0 (34–1677)	
PA classification (cases)		
Aldosteronoma	24	
Adrenal cortical hyperplasia	9	
Idiopathic hyperaldosteronism	11	
Aldosteronoma and adrenal cortical hyperplasia	2	
Unidentified	12	

PA: primary aldosteronism; ALD: aldosterone; ADRR: aldosterone-to-renin ratio; ^a^normally distributed data are presented as mean ± SD (range); ^b^data without normal distribution are given as median (range).

**Table 2:** Descriptive representation of 8 false negative cases.

Sex	Age	Aldosterone	Renin	ADRR	Classification
Male	33	197.81	12.94	15.29	Aldosteronoma
Male	48	192.57	13.10	14.70	Hyperplasia
Male	50	615.38	46.60	13.21	Aldosteronoma
Female	56	501.82	18.40	27.27	Aldosteronoma
Male	38	202.10	7.80	25.91	Unidentified
Female	28	1116.09	61.40	18.18	Unidentified
Male	51	254.33	11.70	21.74	Hyperplasia
Male	42	1000.00	353.70	2.83	Aldosteronoma

**Table 3:** Diagnostic values of different ADRRs for PA screening.

ADRR	Accuracy (%)	False positive rate (%)	False negative rate (%)	Sensitivity (%)	Specificity (%)	Youden
26.0	83.61	17.78	12.07	87.93	82.22	0.7015
28.3	86.13	13.89	13.79	86.21	86.11	0.7232
30.0	86.97	12.22	15.52	84.48	87.78	0.7226
32.0	87.82	9.44	20.69	79.31	90.56	0.6987

## Data Availability

The data used to support the findings of this study are available from the corresponding author upon request.

## Supplementary Materials

Supplementary Materials Supplementary Table 1: diagnostic values of ARR in different patient sets.
