# Supplementary material for: High Efficiency and Problems of Chemiluminescence Assay-Detected Aldosterone-To-Renin Ratio in Practical Primary Aldosteronism Screening
Source: Int J Hypertens. 2020 Aug 27;2020:3934212. doi: 10.1155/2020/3934212 (PMC7474363; doi:10.1155/2020/3934212)
Supplement: Supplementary Materials — Supplementary Table 1: diagnostic values of ARR in different patient sets. [file 3934212.f1.docx]

Supplementary table 1 Diagnostic values of ARR in different patient sets

| Patient | Accuracy | False positive rate | False negative rate | Sensitivity | Specificity |
| --- | --- | --- | --- | --- | --- |
| All | 86.13% | 13.89% | 13.79% | 86.21% | 86.11% |
| Without IGT | 86.70% | 13.29% | 13.33% | 86.67% | 86.71% |

IGT: impaired glucose tolerance.
